# Supplementary material for: Splice-Junction-Based Mapping of Alternative Isoforms in the Human Proteome
Source: Cell Rep. Author manuscript; Available in PMC 2020 Jan 15. (PMC6961840; doi:10.1016/j.celrep.2019.11.026)
Supplement: 2 [file NIHMS1546469-supplement-2.zip › DF1/PXD010154/Thyroid/Thyroid_23_SCRIB_GLGPVPPHAWACSLPGAAVLPGAAEVLCNR.pdf]

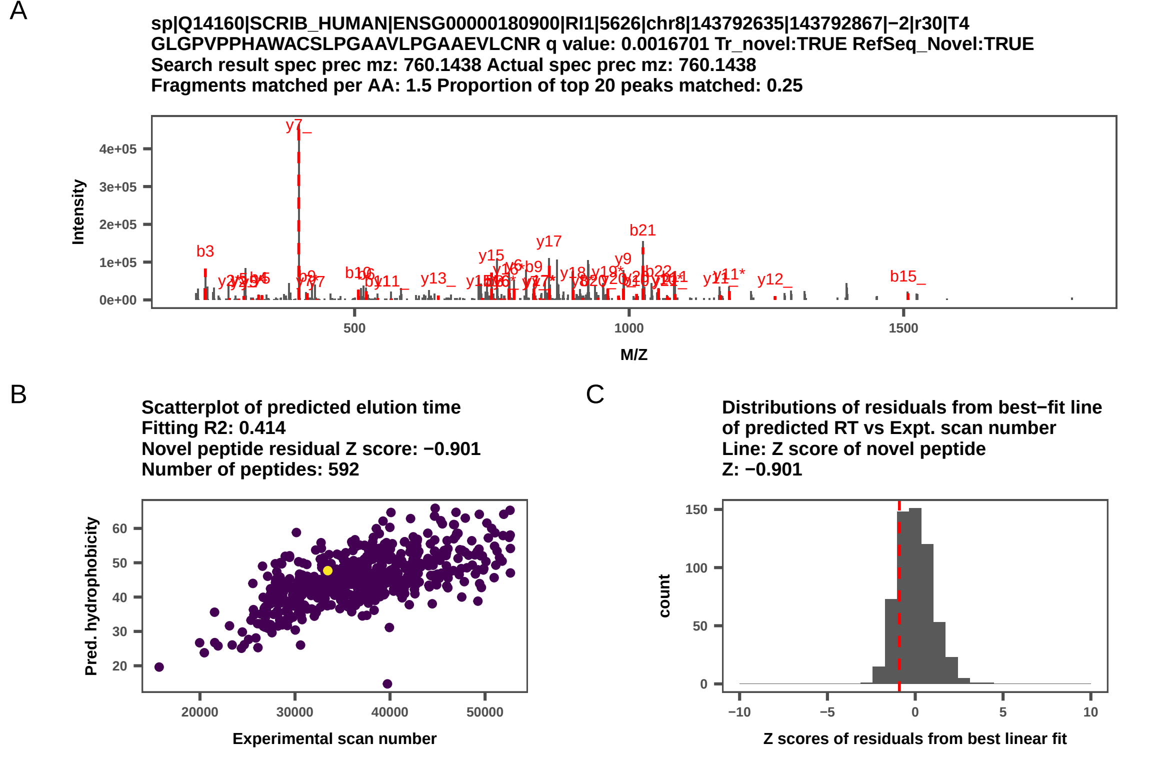
A
sp|Q14160|SCRIB_HUMAN|ENSG00000180900|RI1|5626|chr8|143792635|143792867|−2|r30|T4
GLGPVPPHAWACSLPGAAVLPGAAEVLCNR q value: 0.0016701 Tr_novel:TRUE RefSeq_Novel:TRUE
Search result spec prec mz: 760.1438 Actual spec prec mz: 760.1438
Fragments matched per AA: 1.5 Proportion of top 20 peaks matched: 0.25
Intensity
4e+05
3e+05
2e+05
1e+05
0e+00
y7_
b3
b21
y17
y15
y9
y13_
y12_
b15_
500
1000
1500
M/Z
B
Scatterplot of predicted elution time
Fitting R2: 0.414
Novel peptide residual Z score: −0.901
Number of peptides: 592
Pred. hydrophobicity
60
50
40
30
20
20000
30000
40000
50000
Experimental scan number
C
Distributions of residuals from best−fit line
of predicted RT vs Expt. scan number
Line: Z score of novel peptide
Z: −0.901
count
150
100
50
0
−10
−5
0
5
10
Z scores of residuals from best linear fit
